# Effect of Chemical Agents on the Morphology and Chemical Structures of Microplastics

**DOI:** 10.3390/polym14204353

**Published:** 2022-10-15

**Authors:** Hak Bong Lee, Kyong Sub Lee, Seok Jun Kim, Byung Il Choi, Byung Rye Go, Chan Joo Rhu, Tae Hee Han

**Affiliations:** 1 Department of Organic and Nano Engineering, Hanyang University, Seoul 04763, Korea; 2 Materials & Components Research Institute, Korea Testing & Research Institute, Gwacheon-si 13810, Korea; 3 Products Conformity Center, Korea Conformity Laboratories, Seoul 06711, Korea; 4 Consumer Product Center, Korea Conformity Laboratories, Seoul 06711, Korea; 5 The Research Institute of Industrial Science, Hanyang University, Seoul 04763, Korea

**Keywords:** microplastic, chemical resistance, chemical digestion, degradation

## Abstract

Increased demand for plastics leads to a large amount of plastic manufacturing, which is accompanied by inappropriate disposal of plastics. The by-products of these waste plastics are microplastics (MPs; less than 5 nm in size), which are produced because of various environmental and physicochemical factors, posing hazardous effects to the ecosystem, such as the death of marine organisms due to the swallowing of plastic specks of no nutritional value. Therefore, the collection, preparation, identification, and recycling of these microsized plastics have become imperative. The pretreatment of MPs requires numerous chemical agents comprising strong acids, bases, and oxidizing agents. However, there is limited research on the chemical resistance of various MPs to these substances to date. In this study, the chemical resistance of five species of MPs (high-density polyethylene, low-density polyethylene, polystyrene, polyethylene terephthalate, and polypropylene) to sulfuric acid, hydrochloric acid, hydrogen peroxide, potassium hydroxide, and sodium hydroxide was studied. The MPs were reacted with these chemical reagents at preset temperatures and durations, and variations in morphology and chemical structures were detected when the MPs were reacted with mineral acids, such as sulfuric acid. The data pertaining to these changes in MP properties could be a significant reference for future studies on MP pretreatment with strong acids, bases, and oxidizing agents.

## 1. Introduction

Industrialization over the last century has led to increased demand for and production of plastic materials [1,2]. However, poor disposal and the resultant fragmentation of plastics has produced small plastic particles, commonly referred to as microplastics (MPs) with sizes <5 mm [3]. Plastic fragmentation can be caused by a combination of environmental and physicochemical factors, such as mechanical abrasion, ultraviolet (UV) radiation, and others, resulting in numerous secondary MPs [4]. These resultant microscopic plastics are ubiquitously found in marine and fresh water and terrestrial ecosystems across the globe, resulting in biotic interactions [5,6,7,8]. These MPs do not immediately affect living organisms, but long-term exposure causes numerous hazardous effects by various mechanisms, such as intake of toxic additives, inflammation in organisms through sharp edges of MPs, and the resultant translocations in blood circulation [7]. Therefore, sample collections from water, sediment, and biological samples are conducted for identification and analysis of these MPs for further research [9,10]. These sample collections are then followed by sequential sample preparation utilizing a variety of techniques, including density separation, sieving, and digestion [11,12].

Density separation is a useful tool for extracting MPs (0.8–1.6 g·cm^−3^) from beach sands and bottom sediments (2.7 g·cm^−3^) using a salt-saturated solution of density greater than 1.4 g·cm^−3^ [13,14,15,16,17,18]. However, this method cannot achieve separation of MPs from biological tissue and has negligible impact on high-density polymers [19]. Alternatively, chemical digestion procedures utilizing wet peroxide oxidation (oxidizing agents), alkaline, or acidic conditions have been extensively used to degrade natural organic matter and separate MPs, which have chemical resistance to various chemical agents. The National Oceanic and Atmospheric Administration (Silver Spring, MD, USA; NOAA) recommends using a mixture of H_2_O_2_ (30%) and Fe(II) solution (0.05 M) as a digestion agent, and addition, modified versions of this approach have been extensively investigated [20]. As follows, for acidic digestion, nitric acid (HNO_3_, 55%) at 80 °C and hydrochloric acid (HCl, 37%) have been reported to destroy organic matter (biologic materials such as fish tissue) attached to MPs [14]. As an alternative to acidic digestion, alkaline digestion has been studied: treatments with KOH (10%) and NaOH at 60 °C with a predetermined digestion duration were reported to be effective for digestion [21,22]. In addition, acid and alkali agents (e.g., NaOH and HNO_3_) were concurrently used for yielding effective digestion [23]. Hydrogen peroxide (H_2_O_2_, 30–35%), as an oxidizing agent at 25 °C and 50 °C, was also reported to digest organic matter with high efficiency [22,24]. This peroxide combined with mineral acids, such as sulfuric acid, was used as an effective digestion agent for MPs [25]. However, the use of strong acids, bases, and oxidizing agents for digestion causes fragments and destroys several types of synthetic polymers, including melting and discoloration; therefore, enzymatic digestion, which is less hazardous and less likely to cause damage to MPs, was considered an alternative to chemical digestion. Nevertheless, owing to the high cost of enzymes, even for small-scale operations, enzymatic digestion has a critical limit and requires posttreatment with H_2_O_2_ for removing the remaining organic matter [26].

Despite their wide utilization for pretreatment or chemical digestion, the changes induced by the various chemical agents, such as H_2_O_2_, and strong acids and bases, in the chemical properties of MPs have not been examined in detail. Extensively researched data on the variations in chemical structures and the resultant changes in MP morphologies can be used as the basis for improved MP detection and analysis following pretreatment in the future. Therefore, in this study, the impact of acidic and alkaline agents on the morphology and chemical structure of MP samples with a size range of 20–70 μm was examined. High-density polyethylene (HDPE), low-density polyethylene (LDPE), polypropylene (PP), polystyrene (PS), and polyethylene terephthalate (PET) were selected as the MP polymers because they are predominantly (70% of all polymers) present in the environment [27]. To test their chemical stability, polymer MP samples were processed by sequential ultrafine particle grinding from polymer pellets and resins, followed sieve shaking to control the sizes of the sample to approximately 20 μm. Sulfuric acid (H_2_SO_4_), hydrochloric acid (HCl), hydrogen peroxide (H_2_O_2_), potassium hydroxide (KOH), and sodium hydroxide (NaOH) were selected as chemical agents to react with MP polymers to explore the chemical resistance of these species. Reaction conditions (temperature and reaction duration), posttreatment after reaction with chemical agents, and storage of MPs were controlled to enhance data reliability. For detecting morphological changes in the MPs, scanning electron microscopy (SEM) was employed. Functional groups of the MP polymers before and after the reactions with the chemical agents were observed by Fourier-transform infrared (FT-IR) spectroscopy. Nuclear magnetic resonance (NMR) was selected to investigate molecular structure and chemical composition variations in the MP samples after the reaction. All analytical data were cross-validated to be proposed as the new reference data for the physical and chemical variations induced by the acidic and alkaline pretreatments. The results presented in this manuscript will be the foundation stone for researchers who are focusing on the preparation and pretreatment processes of MPs and the contamination and risks of MPs in the environment.

## 2. Materials and Methods

### 2.1. Materials

HDPE, LDPE, PS, PP, and PET MPs with average sizes ranging from 20 to 70 µm were prepared by the following steps. First, the polymer pellets were immersed in liquid nitrogen and then ground by the ultrafine particle grinder to produce fine polymer-based MPs. Then, the MPs were passed through a stack of sieves of pore sizes 106, 75, 45, and 20 μm. The morphologies of the MP samples obtained via SEM are shown in Figures S1–S5. The size distributions of the HDPE, LDPE, PS, PET, and PP MP samples were 17.5–71.5 μm (Figure S1), 11.1–31.9 μm (Figure S2), 7.9 to 32.1 μm (Figure S3), 11.4–35.4 μm (Figure S4), and 11.9–37.8 μm (Figure S5), respectively. A majority of the microparticle sizes ranged from 17.5 to 25 μm. HDPE (M850 grade) was obtained from Korea Petrochemical Ind. Co., Ltd. (Seoul, Korea). PS (GP 150E grade) was procured from Kumho Petrochemical Co., Ltd. (Seoul, Korea). LDPE (830 grade) was purchased from Hanwha Solutions Co., Ltd. (Seoul, Korea). PET (COOL grade) was procured from Lotte Chemical Co., Ltd. (Seoul, Korea). PP (HJ500 grade) was obtained from Hanwha TotalEnergies Petrochemical Co., Ltd. (Seoul, Korea). The ultrafine particle grinder (DSCH-750) was obtained from Duksan Co., Ltd. (Gwangju, Korea). The sieve shaker (ANALYSETTE 3 SPARTAN) was purchased from FRITSCH Milling and Sizing Inc. (Weimar, Germany). Junsei Chemical Co. Ltd. (Tokyo, Japan) supplied concentrated sulfuric acid (95 wt.% in water, guaranteed reagent (GR) grade), hydrochloric acid (37 wt.% in water, GR grade), and hydrogen peroxide (30 wt.% in water, extra pure grade). The concentrated sulfuric acid was refrigerated and all the reagents were utilized exactly as received. Potassium hydroxide was purchased from Samchun Chemicals Co., Ltd. (Seoul, Korea). Sodium hydroxide was supplied by Junsei Chemical Co., Ltd. (Tokyo, Japan). Ultrapure deionized water (DIW, electrical resistivity of 18.2 MΩ·cm) was obtained from the Direct Q3 system (Millipore Inc., St. Louis, MO, USA). All chemicals were utilized without any additional purification.

### 2.2. Experiment Procedure of Chemical Reactions of Microplastics with Acids, Bases, and Oxidizing Agents

Five sets of 0.5 g of all the MP samples were weighed and loaded in 20 mL vials. Then, 10 mL of the acids, bases, and oxidizing agents were added to each sample set for the reaction with MP samples. The reactions were conducted at 25 and 70 °C to investigate the effects of temperature on the property modification of the polymers. After one and seven days of reactions between the MPs and acids, bases and oxidizing agents, the mixtures were washed several times by DIW using a vacuum-assisted filtration kit to remove the remaining acids, bases, and oxidizing agents. The washed MP powders were dried overnight and stored in a desiccator for further characterization. The acids and oxidizing agent were used without any dilution. For chemical stability tests, 1 M of potassium hydroxide and sodium hydroxide solution were synthesized.

### 2.3. Characteristics of MP Samples before and after Exposure to the Acids, Bases, and Oxidizing Agents

SEM was performed using a JSM-7800 Prime microscope (JEOL Ltd., Tokyo, Japan). The functionalities of the various MP samples before and after exposure to the acids, bases, and oxidizing agents were identified by ATR (attenuated total reflection) detecting mode of Fourier-transform infrared spectroscopy (FT-IR, TENSOR27, Bruker, Borken, Germany). The detection range was 4000–400 cm^−1^, the wavenumber accuracy was 0.01 cm^−1^, and the resolution was 0.4 cm^−1^. ^13^C NMR measurements were conducted on an Advanced III HD Solid-state NMR spectrometer (Bruker, Germany). Solid-state ^13^C CPTOSS NMR spectra were obtained using 4 mm C Cross-Polarisation Magic-Angle Spinning (CPMAS) probes and a 500 MHz Advanced III HD NMR spectrometer. A spinning speed of 5 kHz and pulse repetition delays of 5 s were utilized.

## 3. Results

### 3.1. Chemical Stability Tests of Microplastic Samples

**Figure 1:**
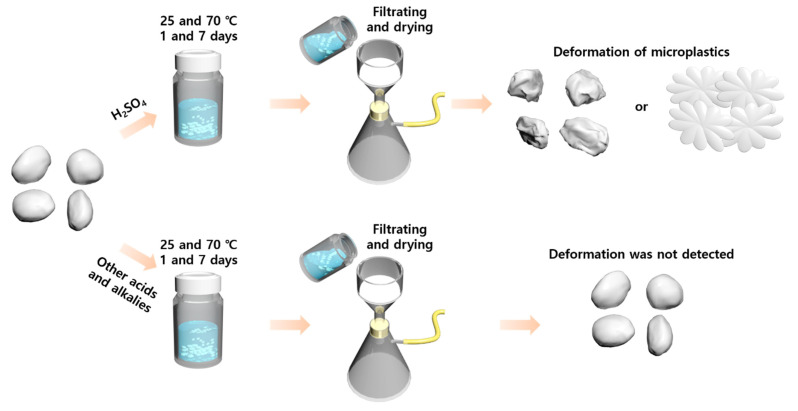
Schematic of the chemical stability test procedure for the microplastic samples (HDPE, LDPE, PP, PET, and PS) against chemical agents (H_2_SO_4_, HCl, H_2_O_2_, KOH, and NaOH) used in the chemical digestion procedure.

To evaluate the chemical stability, the MP samples obtained from all the polymer pellets and resins are immersed in the solutions of acid, alkali, and oxidizing agent in 20 mL vials (Figure 1). The reaction of the polymer microparticles with the strong acids, bases, and oxidizing agent were performed for one and seven days at 25 and 70 °C. After 1 and 7 d, the reacting MP samples were repeatedly filtrated and washed by ultrapure deionized water until the remaining chemicals were completely removed. The washed MP samples were dried over 24 h and stored in a desiccator wrapped with aluminum foil to protect the MPs from ultraviolet light for further chemical characterization and observation of sample morphologies.

### 3.2. Chemical Stability of HDPE against Acids, Bases, and Oxidizing Agents

**Figure 2:**
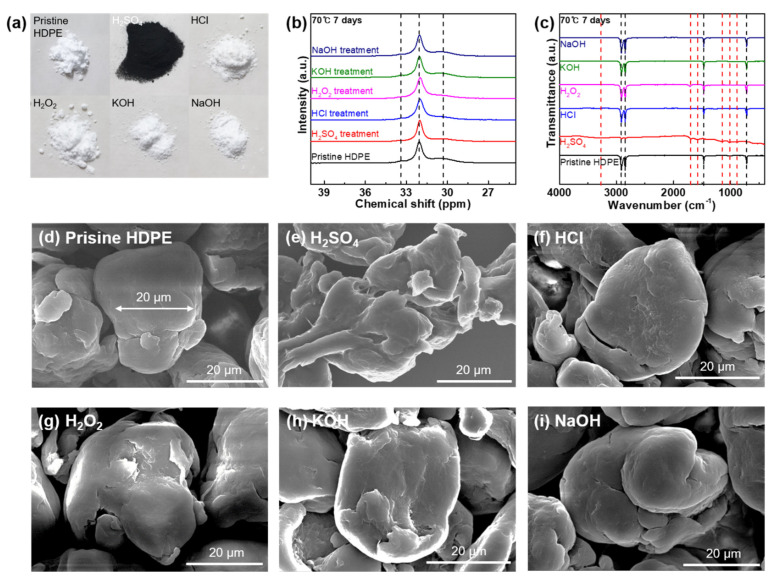
(**a**) Photograph of the HDPE samples in the pristine state and after chemical stability tests against strong acids, bases, and oxidizing agents (H_2_SO_4_, HCl, H_2_O_2_, KOH, and NaOH) used in the chemical digestion procedure at 70 °C for 7 d. (**b**) ^13^C NMR spectra of HDPE in the pristine state and after the reaction with all the chemical agents at 70 °C for 7 d. (**c**) FT-IR spectra of HDPE in the pristine state and after the reaction with all the chemical agents at 70 °C for 7 d. (**d**) SEM image of pristine HDPE microparticles. SEM images of HDPE microparticles after the reaction with (**e**) H_2_SO_4_, (**f**) HCl, (**g**) H_2_O_2_, (**h**) KOH, and (**i**) NaOH at 70 °C for 7 d.

Figure 2 shows the changes in colors, functionalities, carbon structures, and morphologies of HDPE MPs after the reaction with acids, bases, and oxidizing agents. After completing the chemical stability testing procedures, including washing and drying, the HDPE MP samples were collected and characterized. As observed with the naked eye, the HDPE MPs reacted with H_2_SO_4_ at 70 °C for 1 and 7 d, resulting in color alterations from white to black, indicating chemical changes (Figure 2a and Figure S6b). However, there was no color change observed for the HDPE MP samples reacted with HCl, H_2_O_2_, KOH, and NaOH (Figure 2a and Figure S6). Solid-state ^13^C NMR and FT-IR characterizations were conducted to monitor the carbon structures and functionalities of the HDPE MPs before and after the chemical stability tests. Because of the monoclinic crystalline component, a minor resonance signal at 33.4 ppm can be detected in Figure 2b and Figure S7a–c for all the HDPE MP samples [28,29]. A strong resonance signal at 32.0 ppm is ascribed to the orthorhombic crystalline component of the HDPE MPs. The weak signal at 30.3 ppm is assigned to the non-crystalline component of the HDPE MPs [28,29]. After the reaction with the chemical agents, there were no changes or new signals in the spectra of any of the HDPE MP samples, indicating that the carbon structures of the HDPE MP samples were not altered. Figure 2c and Figure S7d–f depict a series of attenuated total reflection (ATR) spectra of the HDPE MP samples prior to and following the chemical reactions with all the chemical agents at all predetermined times and temperatures. For the pristine HDPE MPs, the bands at 2913.9, 2845.9, 1471.9, and 717.4 cm^−1^ denote CH_2_ asymmetric stretching, CH_2_ symmetric stretching, CH_3_ bending deformation, and CH_2_ rocking deformation, respectively [30]. Further, consistency in the FT-IR ATR spectra suggests the HDPE MPs were stable against HCl, H_2_O_2_, KOH, and NaOH. In contrast to the other chemical agents, the HDPE MPs that were exposed to H_2_SO_4_ exhibited several new bands, indicating surface sulfonation. At 25 °C for 1 and 7 d, new bands assigned to S–O–C stretching (888.6 cm^−1^), O=S=O stretching (1024.1 and 1159.4 cm^−1^), C=O stretching (1697.1 cm^−1^), and O–H stretching (3271.6 cm^−1^) [31] were observed. At 70 °C for 1 and 7 d, the peak at 1577.6 cm^−1^ can be attributed to C=C stretching in addition to the newly generated peaks [31]. Figure 2d depicts an SEM image of an HDPE MP sample in its virgin state. The HDPE MP sample exhibits a near-spherical shape with a diameter of approximately 25 μm, as demonstrated in Figure 2d,e–i and Figure S8, and depict the morphologies of the HDPE MP samples that reacted with the acids, bases, and oxidizing agent at predetermined temperature and reaction duration. Surface deformations of the MP particles were detected as observed in Figure 2e and Figure S8a,f,k, indicating the chemical reactions occurring between HDPE and H_2_SO_4_ regardless of temperature and reaction duration. However, when the HDPE MP samples were reacted with other acid, bases, and oxidizing agents, morphological deformation is not observed, indicating that the HDPE MP particles are chemically stable in HCl, H_2_O_2_, KOH, and NaOH regardless of temperature and reaction time (Figure 2f–i and Figure S8).

### 3.3. Chemical Stability of LDPE against Acids, Alkalis, and Oxidizing Agents

**Figure 3:**
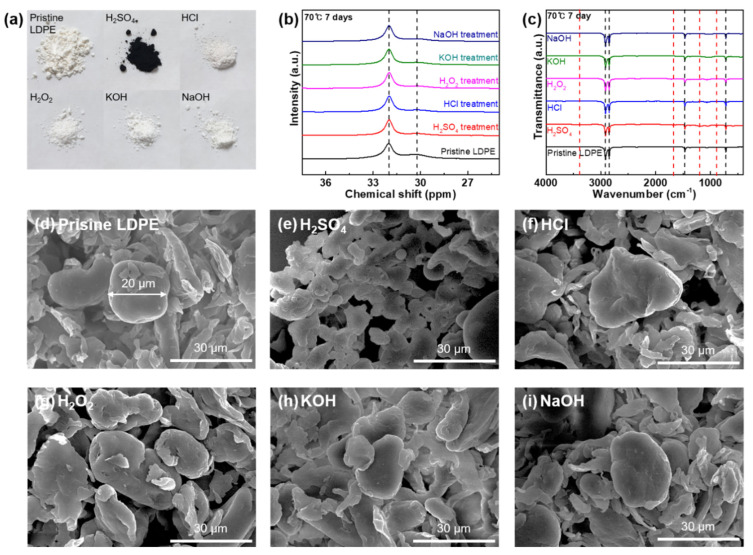
(**a**) Photograph of the LDPE samples in the pristine state and after chemical stability tests against strong acids, bases, and oxidizing agents (H_2_SO_4_, HCl, H_2_O_2_, KOH, and NaOH) used in the chemical digestion procedure at 70 °C for 7 d. (**b**) ^13^C NMR spectra of LDPE in the pristine state and after the reaction with all the chemical agents at 70 °C for 7 d. (**c**) FT-IR spectra of LDPE in the pristine state and after the reaction with all the chemical agents at 70 °C for 7 d. (**d**) SEM image of pristine LDPE microparticles. SEM images of the LDPE microparticles after the reaction with (**e**) H_2_SO_4_, (**f**) HCl, (**g**) H_2_O_2_, (**h**) KOH, and (**i**) NaOH at 70 °C for 7 d.

Figure 3 displays the changes in colors, functionalities, carbon structures, and morphologies of LDPE MPs after the reaction with acids, bases, and oxidizing agents. The LDPE MP samples were collected and analyzed in the same manner as the HDPE MP samples following all the chemical stability tests. After the reaction with H_2_SO_4_ at 70 °C after 1 and 7 d, the LDPE MP samples exhibit a color change from white to brown and black, respectively (Figure 3a and Figure S9b). Figure 3a and Figure S9 reveal that, similarly to the color change observed in the HDPE MP samples, the LDPE MP samples exposed to the various chemical agents did not exhibit a color change. Similarly, solid-state ^13^C NMR and FT-IR spectra were obtained to observe carbon structures and functionalities of the LDPE MPs before and after the chemical stability tests. In Figure 3b and Figure S10a–c, the monoclinic crystalline component, which appears as a minor resonance signal at 33.4 ppm, was not detected in the LDPE MP samples. This is unlike the observation in the HDPE MP samples. However, a strong resonance signal at 32.0 ppm owing to the orthorhombic crystalline component of the LDPE MPs is comparable to the result of the HDPE MPs [28,29]. The non-crystalline component from the LDPE MPs at 30.2 ppm appears as a weak signal [28,29]. Moreover, there was no change in the carbon structures of any of the LDPE MPs, suggesting that they are chemically resistant to all the chemical agents tested regardless of preset temperature. This chemical stability is attributed to the main chain of the LDPE samples. In Figure 3c and Figure S10d–f, the functionality changes in the LDPE MP samples subjected to acids, bases, and oxidizing agents are depicted to examine their surface chemical alterations. The pristine LDPE MPs exhibited 2916.1, 2848.5, 1471.5, and 717.4 cm^−1^ of bands, designated as CH_2_ asymmetric stretching, CH_2_ symmetric stretching, CH_3_ bending deformation, and CH_2_ rocking deformation, respectively [30]. Similarly to the results of the HDPE MPs, the LDPE MP exhibited chemical resistance to HCl, H_2_O_2_, KOH, and NaOH with consistent FT-IR spectra at all testing conditions (Figure 3c and Figure S10d–f). With H_2_SO_4_ at 25 °C for one day, no new bands or band shifts in the FT-IR spectrum of the LDPE MP sample are observed, suggesting good chemical stability (Figure S10d). Alternatively, with H_2_SO_4_ at 25 °C for 7 d and 70 °C for 1 and 7 d, a chemical functionality change on the surface of the LDPE MPs is observed (Figure 3c and Figure S10e–f). An S–O–C stretching band at 885.2 cm^−1^, O=S=O stretching band at 1189.9 cm^−1^, a C=O stretching band at 1675.9 cm^−1^, and an O–H stretching band at 3394.3 cm^−1^ are observed, demonstrating that H_2_SO_4_ induces sulfonation on the surface of the LDPE MP samples [31]. The morphological changes in the LDPE MP particles detected by SEM were similar to those in HDPE MP particles; the LDPE MP particles are spherical shaped and 20 μm in size (Figure 3d). This is indicative of surface deformation and aggregation when reacted with H_2_SO_4_ at 70 °C for 7 d (Figure 3e). Regardless of temperature and reaction duration conditions, H_2_SO_4_ induced surface deformation in the LDPE microparticles (Figure S11a,f,k). However, the other chemical agents (HCl, H_2_O_2_, KOH, and NaOH) did not affect the morphology of the LDPE MPs (Figure 3f–i and Figure S11).

### 3.4. Chemical Stability of PS against Acids, Bases, and Oxidizing Agents

**Figure 4:**
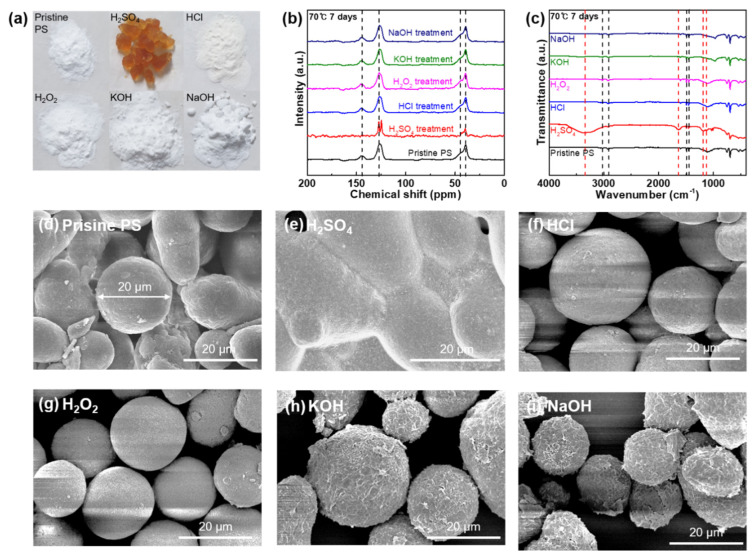
(**a**) Photograph of the PS samples in the pristine state and after chemical stability tests against strong acids, bases, and oxidizing agents (H_2_SO_4_, HCl, H_2_O_2_, KOH, and NaOH) used in the chemical digestion procedure at 70 °C for 7 d. (**b**) ^13^C NMR spectra of PS in the pristine state and after the reaction with all chemical agents at 70 °C for 7 d. (**c**) FT-IR spectra of PS in the pristine state and after the reaction with all chemical agents at 70 °C for 7 d. (**d**) SEM image of the pristine PS microparticles. SEM images of the PS microparticles after the reaction with (**e**) H_2_SO_4_, (**f**) HCl, (**g**) H_2_O_2_, (**h**) KOH, and (**i**) NaOH at 70 °C for 7 d.

Figure 4 presents the changes in colors, functionalities, carbon structures, and morphologies of PS MPs after the reaction with acids, bases, and oxidizing agents. After treating the PS MP samples with the acids, bases, and oxidizing agents, the samples were characterized. In the bulk state, the PS MP samples exhibit a color change from white to orange and swelling with H_2_SO_4_ at 70 °C for 1, and with H_2_SO_4_ at 70 °C and 7 d (Figure 4a and Figure S12b). The PS MP samples exhibit a shift in hue from white to yellow during the HCl reaction at 25 °C for 1 and 7 d (Figure S12a,c). In contrast, the other chemical agents did not affect PS MP samples in the bulk state regardless of temperature and reaction time (Figure 4a and Figure S12). The ^13^C NMR spectra of the PS MP samples show resonance signals at 144.8, 127.4, 45.1, and 39.4 ppm, which were attributed to non-protonated, protonated aromatic carbons, methylene, and methyl carbons of the pristine PS carbon structures, respectively (Figure 4b and Figure S13a–c) [32,33]. The PS MP samples exhibit chemical resistance to HCl, H_2_O_2_, KOH, and NaOH at all preset conditions, indicated by the unchanging ^13^C NMR spectra (no new bands and no chemical shifts). In addition, H_2_SO_4_ at 25 °C for 1 and 7 d cannot derive any chemical reactions to PS MP samples. However, with H_2_SO_4_ at 70 °C for 1 and 7 d, the resonance signals at 144.8, 45.1, and 39.4 ppm were suppressed. Furthermore, the protonated aromatic carbon peak at 127.4 ppm, which is designated as the mono-substituted toluene sulfonic acid signal, was firmly generated [34]. To further investigate the functionality changes in the PS MP samples, FT-IR characterization was conducted before and after the chemical stability tests (Figure 4c and Figure S13d–f). Several bands at 3028.7, 2907.2, 1492.6, and 1451.6 cm^−1^ are observed, denoting aromatic stretching (=C–H), asymmetric stretching (–CH_2_–), and aromatic stretching (C=C), respectively, for the pure PS MPs [35]. As illustrated in Figure 4c and Figure S13d–f, HCl, H_2_O_2_, KOH, and NaOH at all predetermined temperatures and reaction durations did not result in any chemical modifications on the surface of the PS MP samples. The FT-IR spectra of the PS MPs reacted with H_2_SO_4_ at 25 °C for 1 d exhibits chemical resistance to H_2_SO_4_ at room temperature (Figure S13d). The reaction with H_2_SO_4_ at 25 °C for 7 d and 70 °C for 1 and 7 d yield surface sulfonation in the PS MPs, as can be observed in Figure 4c and Figure S13e,f. Surface sulfonation in the PS MPs can be detected by the O–H stretching band at 3337.3 cm^−1^, the C=O stretching band at 1624.8 cm^−1^, the S–O band at 1181.2 cm^−1^, and the O=S=O symmetric stretching band at 1127.2 cm^−1^ [35,36]. SEM observations (Figure 4d–i and Figure S14) of the pristine PS microparticle reveal a round shape with a size of approximately 20 μm (Figure 4d). After the reaction with H_2_SO_4_ at 70 °C for 1 and 7 d, swelling and aggregation are detected, which corresponds to the optical observation (Figure 4e and Figure S14f). In addition, surface deformation was observed in the PS MPs when the particles are reacted with H_2_SO_4_ at 25 °C, and KOH and NaOH at 70 °C for 1 and 7 d (Figure 4h,i and Figure S14i,j). The reaction of the PS MP samples with the other chemical agents under the predetermined conditions did not result in morphological changes (Figure 4f,g and Figure S14).

### 3.5. Chemical Stability of PET against Acids, Alkalis, and Oxidizing Agents

**Figure 5:**
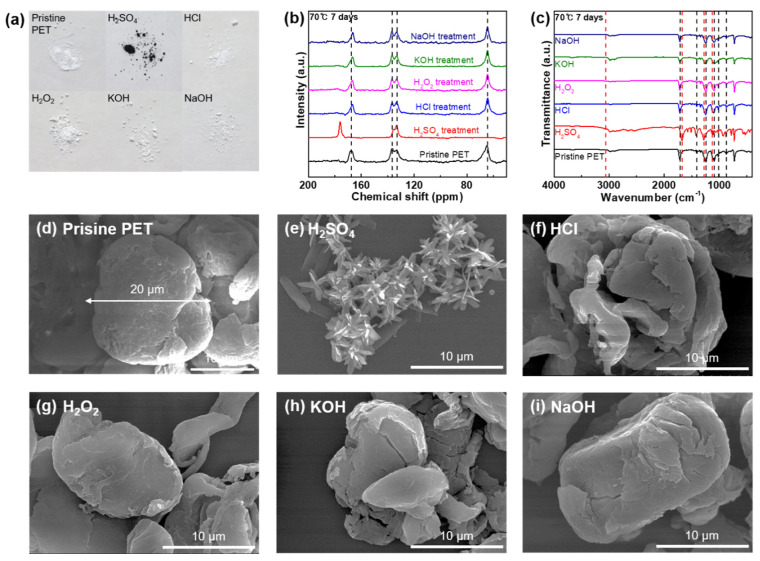
(**a**) Photograph of the PET samples of pristine state and after chemical stability tests against strong acids, bases, and oxidizing agents (H_2_SO_4_, HCl, H_2_O_2_, KOH, and NaOH) used in the chemical digestion procedure at 70 °C for 7 d. (**b**) ^13^C NMR spectra of PET in the pristine state and after the reaction with all the chemical agents at 70 °C for 7 d. (**c**) FT-IR spectra of PET in the pristine state and after the reaction with all the chemical agents at 70 °C for 7 d. (**d**) SEM image of pristine PET microparticles. SEM images of the PET microparticles after the reaction with (**e**) H_2_SO_4_, (**f**) HCl, (**g**) H_2_O_2_, (**h**) KOH, and (**i**) NaOH at 70 °C for 7 d.

Figure 5 illustrates the changes in colors, functionalities, carbon structures, and morphologies of PET MPs after the reaction with acids, bases, and oxidizing agents. The PET MP samples were characterized after being treated with all the chemical agents used in this study. The PET MP samples reacted with H_2_SO_4_ at 70 °C for 1 and 7 d, change color from white to black in the bulk state (Figure 5a and Figure S15b). In contrast, the PET MP samples exhibit chemical resistance to HCl, H_2_O_2_, KOH, and NaOH under all predetermined conditions in the bulk state (Figure 5a and Figure S15). As depicted in ^13^C NMR spectra of pristine PET, signals at 167.4, 136.7, 133.2, and 64.7 ppm were assigned to COO, aromatic C adjacent to the ester, aromatic C, and COOCH_2_, respectively (Figure 5b and Figure S16a–c) [37]. Chemical deformation or degradation was not detected with the treatment of HCl, H_2_O_2_, KOH, and NaOH at any predetermined temperature and treatment duration, demonstrating the chemical resistance of PET to these chemical agents (Figure 5b and Figure S16a–c). However, PET breakdown into terephthalic acid monomer was observed when treated with H_2_SO_4_ (Figure 5b and Figure S16a–c). The signal for carboxylic acid at 172 ppm appears, and the signal for the C adjacent to the ester shifts from 136.7 to 135.5 ppm because the carboxylic esters hydrolyze to carboxylic acid. The ethylene carbon adjacent to the hydroxyl resonance signal (64.7 ppm) disappears because ethylene glycol is eliminated from the material during decomposition [37,38]. This decomposition is not completed with the treatment of H_2_SO_4_ at 25 °C after 1 d (Figure S16a), but harsher predetermined temperatures and longer reaction durations yield a complete conversion of the PET MPs into the terephthalic acid monomer (Figure 5b and Figure S16a–c). The FT-IR ATR spectra of PET before and after the chemical stability test was observed to confirm the chemical resistance of the PET MP samples against the acids, bases, and oxidizing agents (Figure 5c and Figure S16d–f). For the pristine PET MPs, a typical signal at 1714.5 cm^−1^ corresponding to the C=O stretching of ester bonds is confirmed in addition to the C–C phenyl ring band at 1407.8 cm^−1^, and C–O stretching band at 1242.1 and 1093.5 cm^−1^. Two peaks at 1016.3 and 873.7 cm^−1^ were designated as the distinctive peaks of pristine PET (Figure 5c and Figure S16d–f) [39,40]. The PET MP samples are chemically resistant to HCl, H_2_O_2_, KOH, and NaOH, as evidenced by unchanging ATR spectra of the PET MPs (Figure 5c and Figure S16d–f). In contrast, after reaction with H_2_SO_4_ at all temperatures and reaction durations, the ATR spectra of the PET MPs exhibit typical bands were designated as aromatic dicarboxylic acids at 3062.6 cm^−1^ of the =C–H aromatic band, 1677.8 cm^−1^ of the C=O stretching band, 1278.6 cm^−1^ of C–O stretching band, and 1130.1 and 1018 cm^−1^ of the 1,4-disubstituted benzene ring band, illustrating hydrolysis of PET into the terephthalic acid monomer [39,40,41]. The SEM images of the PET MPs following their reaction with all the chemical agents are shown in Figure 5d–i and Figure S17. As depicted in Figure 5e and Figure S17a,f,k after reacting with H_2_SO_4_ at all predetermined conditions, the 20 μm-sized PET MP particles degrade into flower-shaped terephthalic acid. Figure 5f–i and Figure S17 illustrates the chemical resistance of the PET MP samples to different chemical agents including HCl, H_2_O_2_, KOH, and NaOH in all predetermined circumstances.

### 3.6. Chemical Stability of PP against Acids, Bases, and Oxidizing Agents

**Figure 6:**
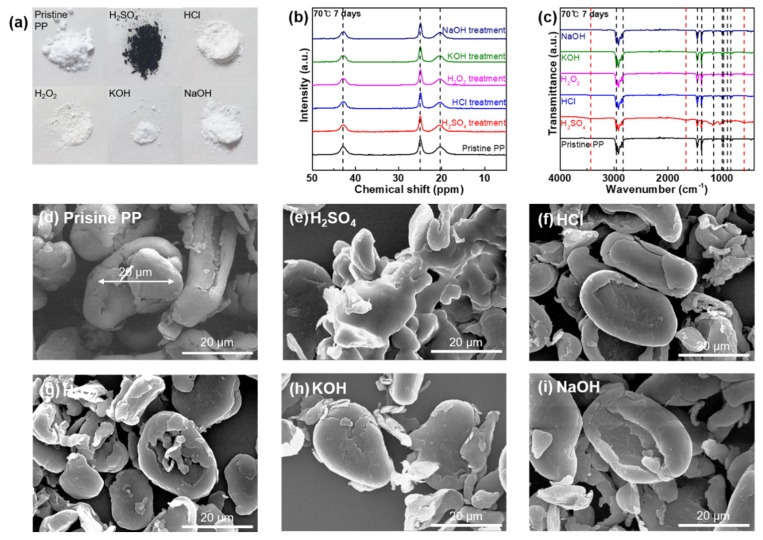
(**a**) Photograph of the PP samples in the pristine state and after chemical stability tests against strong acids, bases, and oxidizing agents (H_2_SO_4_, HCl, H_2_O_2_, KOH, and NaOH) used in the chemical digestion procedure at 70 °C for 7 d. (**b**) ^13^C NMR spectra of PP in the pristine state and after the reaction with all chemical agents at 70 °C for 7 d. (**c**) FT-IR spectra of PP in the pristine state and after the reaction with all chemical agents at 70 °C for 7 d. (**d**) SEM image of pristine PP microparticles. SEM images of PP microparticles after the reaction with (**e**) H_2_SO_4_, (**f**) HCl, (**g**) H_2_O_2_, (**h**) KOH, and (**i**) NaOH at 70 °C for 7 d.

Figure 6 shows the changes in colors, functionalities, carbon structures, and morphologies of PP MPs after the reaction with acids, bases, and oxidizing agents. After the chemical stability test, the PP MP samples were collected and their bulk states were recorded. Similarly to the LDPE MP particles, the PP MPs undergo a color change from white to brown and black after reacting with H_2_SO_4_ at 70 °C for 1 and 7 d, respectively, (Figure 6a and Figure S18b). In addition, the PP MP samples reacted with HCl, H_2_O_2_, KOH, and NaOH do not undergo a color change (Figure 6a and Figure S18). To examine the carbon structure of the PP MP samples, ^13^C NMR spectroscopy was conducted on all the PP MP samples before and after chemical stability testing (Figure 6b and Figure S19a–c). For the pristine PP MPs, resonance signals appear at 42.8, 24.9, and 20.3 ppm, denoting carbon components of–CH_2_–,–CH–, and–CH_3_ in the polymer main chains [42]. No new resonance signals are observed in the ^13^C NMR spectra of the PP MP samples for reactions with any of the chemical agents or under any of the predetermined conditions, demonstrating the chemical resistance of the carbon structures. This is also confirmed by the ATR-FT-IR spectra (Figure 6c and Figure S19d–f). As shown in Figure 6c and Figure S19d–f, the pristine PP MPs exhibit four prominent peaks at 2951.5 and 2868.6 cm^−1^ for CH_3_ asymmetric and symmetric stretching vibrations, and at 2917.3 and 2838.2 cm^−1^ for CH_2_ asymmetric and symmetric stretching vibration bands. In addition, the two peaks at 1456.9 and 1375.7 cm^−1^ are attributed to the CH_3_ asymmetric and symmetric deformation vibrations. The peak at 1167.7 cm^−1^ is designated to the C–C asymmetric stretching vibrations. The peaks at 997.9, 972.9, and 899.2 denote the CH_3_ asymmetric rocking vibrations. Lastly, the peaks at 841.4 and 809.4 are attributed to the CH_2_ rocking vibrations (Figure 6c and Figure S19d–f) [43]. With HCl, H_2_O_2_, KOH, and NaOH, no peak shifts or new peaks are observed owing to the chemical resistance of the PP MPs. Moreover, reactions with H_2_SO_4_ at 25 °C for 1 d did not induce chemical modifications on the PP MP surface. However, the longer reaction duration (7 d) or higher temperature (70 °C for 1 and 7 d) using H_2_SO_4_ can induce surface chemical modifications. The O–H stretching band at 3414.4 cm^−1^, the bands in the 1250–840 cm^−1^ region, and the peak at 582 cm^−1^ are attributed to the SO_3_H groups on the surface of the PP MPs. The peak at 1657.5 cm^−1^ denotes the stretching vibrations of C=O [44]. The surfaces of the approximately 20 μm-sized PP microparticles (Figure 6d) were deformed by H_2_SO_4_ at 70 °C for 1 and 7 d, suggesting a chemical reaction between PP MPs and H_2_SO_4_ (SEM images; Figure 6e and Figure S20f). In contrast, the PP MP samples revealed chemical resistance to H_2_SO_4_ at 25 °C for 1 and 7 d in the case of morphological observation (Figure S20a,k). Furthermore, the chemical reaction conditions involving HCl, H_2_O_2_, KOH, and NaOH are insufficient to induce morphological deformations on the surface of the PP MP samples (Figure 6f–i and Figure S20). The table summarizing the overall effects by the addition of chemical reagents for all MPs is as follows (Table 1).

## 4. Conclusions

In this study, five types of polymer-based MP samples were manufactured and reacted with strong acids, bases, and an oxidizing agent under four preset reaction conditions (reaction duration of 1 and 7 d and temperature of 25 and 70 °C) to investigate their chemical resistance. The HDPE, LDPE, and PP MPs exhibited chemical resistance to HCl, H_2_O_2_, KOH, and NaOH at all reaction conditions, as indicated by the unchanged morphology and chemical structures before and after the chemical stability tests. When treated with H_2_SO_4_, the chemical backbones of these three MPs were retained; however, under the temperature of 70 °C, they demonstrated morphological deformation and surface sulfonation. PS exhibited surface abrasion when reacted with bases at 70 °C, while the bases had no influence on its chemical structure. Only the reaction with H_2_SO_4_ at an elevated temperature (70 °C) induced sulfonation of the PS MPs with morphological swelling and aggregation. In the case of PET MPs, H_2_SO_4_ promotes hydrolysis of the PET MPs into nanoflower shaped terephthalic acid under all reaction conditions, while the other chemical agents could not induce any morphological or chemical changes. In conclusion, based on the results of the chemical stability tests, chemical digestion utilizing mineral acids, such as sulfuric acid, can induce morphological deformation and transformation in the chemical structures. As such, using sulfuric acid during the pretreatment of MPs should be avoided when researchers want to prepare MPs without chemical damaging based on findings on the results of chemical stability tests. Furthermore, chemical structure changes of microsized plastic particles showed almost the same results in their bulk states, such as film, which are reported in the related literature. Thus, these results can contribute to future research on chemical digestion of polymer-based MPs.

## Figures and Tables

**Table 1:** The overall effects by the addition of acids, bases, and oxidizing agent on all the MPs.

	H_2_SO_4_	HCl	H_2_O_2_	KOH	NaOH
**HDPE**	Morphologies, functionalities	No effects	No effects	No effects	No effects
**LDPE**	Morphologies, functionalities	No effects	No effects	No effects	No effects
**PS**	Morphologies, functionalities, carbon structures	No effects	No effects	No effects	No effects
**PET**	Morphologies, functionalities, carbon structures	No effects	No effects	No effects	No effects
**PP**	Morphologies, functionalities	No effects	No effects	No effects	No effects

## Data Availability

The data presented in this study are available on request from the corresponding author.

## Supplementary Materials

The following supporting information can be downloaded at https://www.mdpi.com/article/10.3390/polym14204353/s1. Figure S1. (a) SEM image of HDPE MP model samples and (b) their size distribution graph; Figure S2. (a) SEM image of LDPE MP model samples and (b) their size distribution graph; Figure S3. (a) SEM image of PS MP model samples and (b) their size distribution graph; Figure S4. (a) SEM image of PET MP model samples and (b) their size distribution graph; Figure S5. (a) SEM image of PP MP model samples and (b) their size distribution graph; Figure S6. Photograph of the HDPE samples of pristine state and after chemical stability tests against strong acids and alkalis (H_2_SO_4_, HCl, H_2_O_2_, KOH, and NaOH) used in the chemical digestion procedure at (a) 25 °C for 1 day, (b) 70 °C for 1 day, and (c) 25 °C for 7 days; Figure S7. 13C NMR spectra of pristine HDPE and HDPE samples after reaction with H_2_SO_4_, HCl, H_2_O_2_, KOH, and NaOH at (a) 25 °C for 1 day, (b) 70 °C for 1 day, and (c) 25 °C for 7 days. FT-IR spectra of pristine HDPE and HDPE samples after reaction with H_2_SO_4_, HCl, H_2_O_2_, KOH, and NaOH at (d) 25 °C for 1 day, (e) 70 °C for 1 day, and (f) 25 °C for 7 days; Figure S8. SEM images of HDPE samples after reaction with (a) H_2_SO_4_, (b) HCl, (c) H_2_O_2_, (d) KOH, and (e) NaOH at 25 °C for 1 day. SEM images of HDPE samples after reaction with (f) H_2_SO_4_, (g) HCl, (h) H_2_O_2_, (i) KOH, and (j) NaOH at 70 °C for 1 day. SEM images of HDPE samples after reaction with (k) H_2_SO_4_, (l) HCl, (m) H_2_O_2_, (n) KOH, and (o) NaOH at 25 °C for 7 days; Figure S9. Photograph of the LDPE samples of pristine state and after chemical stability tests against strong acids and alkalis at (a) 25 °C for 1 day, (b) 70 °C for 1 day, and (c) 25 °C for 7 days; Figure S10. ^13^C NMR spectra of pristine LDPE and LDPE samples after reaction with H_2_SO_4_, HCl, H_2_O_2_, KOH, and NaOH at (a) 25 °C for 1 day, (b) 70 °C for 1 day, and (c) 25 °C for 7 days. FT-IR spectra of pristine LDPE and LDPE samples after reaction with H_2_SO_4_, HCl, H_2_O_2_, KOH, and NaOH at (d) 25 °C for 1 day, (e) 70 °C for 1 day, and (f) 25 °C for 7 days; Figure S11. SEM images of LDPE samples after reaction with (a) H_2_SO_4_, (b) HCl, (c) H_2_O_2_, (d) KOH, and (e) NaOH at 25 °C for 1 day. SEM images of LDPE samples after reaction with (f) H_2_SO_4_, (g) HCl, (h) H_2_O_2_, (i) KOH, and (j) NaOH at 70 °C for 1 day. SEM images of LDPE samples after reaction with (k) H_2_SO_4_, (l) HCl, (m) H_2_O_2_, (n) KOH, and (o) NaOH at 25 °C for 7 days; Figure S12. Photograph of the PS samples of pristine state and after chemical stability tests against strong acids and alkalis at (a) 25 °C for 1 day, (b) 70 °C for 1 day, and (c) 25 °C for 7 days; Figure S13. 1^3^C NMR spectra of pristine PS and PS samples after reaction with H_2_SO_4_, HCl, H_2_O_2_, KOH, and NaOH at (a) 25 °C for 1 day, (b) 70 °C for 1 day, and (c) 25 °C for 7 days. FT-IR spectra of pristine PS and PS samples after reaction with H_2_SO_4_, HCl, H_2_O_2_, KOH, and NaOH at (d) 25 °C for 1 day, (e) 70 °C for 1 day, and (f) 25 °C for 7 days; Figure S14. SEM images of PS samples after reaction with (a) H_2_SO_4_, (b) HCl, (c) H_2_O_2_, (d) KOH, and (e) NaOH at 25 °C for 1 day. SEM images of PS samples after reaction with (f) H_2_SO_4_, (g) HCl, (h) H_2_O_2_, (i) KOH, and (j) NaOH at 70 °C for 1 day. SEM images of PS samples after reaction with (k) H_2_SO_4_, (l) HCl, (m) H_2_O_2_, (n) KOH, and (o) NaOH at 25 °C for 7 days; Figure S15. Photograph of the PET samples of pristine state and after chemical stability tests against strong acids and alkalis at (a) 25 °C for 1 day, (b) 70 °C for 1 day, and (c) 25 °C for 7 days; Figure S16. 13C NMR spectra of pristine PET and PET samples after reaction with H_2_SO_4_, HCl, H_2_O_2_, KOH, and NaOH at (a) 25 °C for 1 day, (b) 70 °C for 1 day, and (c) 25 °C for 7 days. FT-IR spectra of pristine PET and PET samples after reaction with H_2_SO_4_, HCl, H_2_O_2_, KOH, and NaOH at (d) 25 °C for 1 day, (e) 70 °C for 1 day, and (f) 25 °C for 7 days; Figure S17. SEM images of PET samples after reaction with (a) H_2_SO_4_, (b) HCl, (c) H_2_O_2_, (d) KOH, and (e) NaOH at 25 °C for 1 day. SEM images of PET samples after reaction with (f) H_2_SO_4_, (g) HCl, (h) H_2_O_2_, (i) KOH, and (j) NaOH at 70 °C for 1 day. SEM images of PET samples after reaction with (k) H_2_SO_4_, (l) HCl, (m) H_2_O_2_, (n) KOH, and (o) NaOH at 25 °C for 7 days; Figure S18. Photograph of the PP samples of pristine state and after chemical stability tests against strong acids and alkalis at (a) 25 °C for 1 day, (b) 70 °C for 1 day, and (c) 25 °C for 7 days; Figure S19. 13C NMR spectra of pristine PP and PP samples after reaction with H_2_SO_4_, HCl, H_2_O_2_, KOH, and NaOH at (a) 25 °C for 1 day, (b) 70 °C for 1 day, and (c) 25 °C for 7 days. FT-IR spectra of pristine PP and PP samples after reaction with H_2_SO_4_, HCl, H_2_O_2_, KOH, and NaOH at (d) 25 °C for 1 day, (e) 70 °C for 1 day, and (f) 25 °C for 7 days; Figure S20. SEM images of PP samples after reaction with (a) H_2_SO_4_, (b) HCl, (c) H_2_O_2_, (d) KOH, and (e) NaOH at 25 °C for 1 day. SEM images of PP samples after reaction with (f) H_2_SO_4_, (g) HCl, (h) H_2_O_2_, (i) KOH, and (j) NaOH at 70 °C for 1 day. SEM images of PP samples after reaction with (k) H_2_SO_4_, (l) HCl, (m) H_2_O_2_, (n) KOH, and (o) NaOH at 25 °C for 7 days.

## References

[1] Rillig M.C. (2012). Microplastic in terrestrial ecosystems and the soil?. Environ. Sci. Technol..

[2] Barnes D.K.A., Galgani F., Thompson R.C., Barlaz M. (2009). Accumulation and fragmentation of plastic debris in global environments. Philos. Trans. R. Soc. B Biol. Sci..

[3] Napper I.E., Bakir A., Rowland S.J., Thompson R.C. (2015). Characterisation, quantity and sorptive properties of microplastics extracted from cosmetics. Mar. Pollut. Bull..

[4] Cole M., Lindeque P., Halsband C., Galloway T.S. (2011). Microplastics as contaminants in the marine environment: A review. Mar. Pollut. Bull..

[5] Duis K., Coors A. (2016). Microplastics in the aquatic and terrestrial environment: Sources (with a specific focus on personal care products), fate and effects. Environ. Sci. Eur..

[6] Lusher A.L., Welden N.A., Sobral P., Cole M. (2017). Sampling, isolating and identifying microplastics ingested by fish and invertebrates. Anal. Methods.

[7] Li J., Liu H., Paul Chen J. (2018). Microplastics in freshwater systems: A review on occurrence, environmental effects, and methods for microplastics detection. Water Res..

[8] Eerkes-Medrano D., Thompson R.C., Aldridge D.C. (2015). Microplastics in freshwater systems: A review of the emerging threats, identification of knowledge gaps and prioritisation of research needs. Water Res..

[9] Suaria G., Avio C.G., Mineo A., Lattin G.L., Magaldi M.G., Belmonte G., Moore C.J., Regoli F., Aliani S. (2016). The Mediterranean plastic soup: Synthetic polymers in Mediterranean surface waters. Sci. Rep..

[10] Van Cauwenberghe L., Vanreusel A., Mees J., Janssen C.R. (2013). Microplastic pollution in deep-sea sediments. Environ. Pollut..

[11] Quinn B., Murphy F., Ewins C. (2017). Validation of density separation for the rapid recovery of microplastics from sediment. Anal. Methods.

[12] Zobkov M.B., Esiukova E.E. (2018). Microplastics in a marine environment: Review of methods for sampling, processing, and analyzing microplastics in water, bottom sediments, and coastal deposits. Oceanology.

[13] Prata J.C., da Costa J.P., Duarte A.C., Rocha-Santos T. (2019). Methods for sampling and detection of microplastics in water and sediment: A critical review. TrAC-Trends Anal. Chem..

[14] Munno K., Helm P.A., Jackson D.A., Rochman C., Sims A. (2018). Impacts of temperature and selected chemical digestion methods on microplastic particles. Environ. Toxicol. Chem..

[15] Corcoran P.L., Norris T., Ceccanese T., Walzak M.J., Helm P.A., Marvin C.H. (2015). Hidden plastics of Lake Ontario, Canada and their potential preservation in the sediment record. Environ. Pollut..

[16] Ballent A., Corcoran P.L., Madden O., Helm P.A., Longstaffe F.J. (2016). Sources and sinks of microplastics in Canadian Lake Ontario nearshore, tributary and beach sediments. Mar. Pollut. Bull..

[17] Nuelle M.T., Dekiff J.H., Remy D., Fries E. (2014). A new analytical approach for monitoring microplastics in marine sediments. Environ. Pollut..

[18] Hidalgo-Ruz V., Gutow L., Thompson R.C., Thiel M. (2012). Microplastics in the marine environment: A review of the methods used for identification and quantification. Environ. Sci. Technol..

[19] Claessens M., Van Cauwenberghe L., Vandegehuchte M.B., Janssen C.R. (2013). New techniques for the detection of microplastics in sediments and field collected organisms. Mar. Pollut. Bull..

[20] Masura J., Baker J., Foster G., Authur C. (2015). Laboratory Methods for the Analysis of Microplastics in the Marine environment: Recommendations for Quantifying Synthetic Particles in Waters and Sediments. NOAA Tech. Memo. NOS-OR&R.

[21] Foekema E.M., De Gruijter C., Mergia M.T., Van Franeker J.A., Murk A.J., Koelmans A.A. (2013). Plastic in north sea fish. Environ. Sci. Technol..

[22] Cole M., Webb H., Lindeque P.K., Fileman E.S., Halsband C., Galloway T.S. (2014). Isolation of microplastics in biota-rich seawater samples and marine organisms. Sci. Rep..

[23] Roch S., Brinker A. (2017). Rapid and Efficient Method for the Detection of Microplastic in the Gastrointestinal Tract of Fishes. Environ. Sci. Technol..

[24] Avio C.G., Gorbi S., Regoli F. (2015). Experimental development of a new protocol for extraction and characterization of microplastics in fish tissues: First observations in commercial species from Adriatic Sea. Mar. Environ. Res..

[25] Imhof H.K., Ivleva N.P., Schmid J., Niessner R., Laforsch C. (2013). Contamination of beach sediments of a subalpine lake with microplastic particles. Curr. Biol..

[26] Catarino A.I., Thompson R., Sanderson W., Henry T.B. (2017). Development and optimization of a standard method for extraction of microplastics in mussels by enzyme digestion of soft tissues. Environ. Toxicol. Chem..

[27] Reimonn G., Lu T., Gandhi N., Chen W.T. (2019). Review of microplastic pollution in the environment and emerging recycling solutions. J. Renew. Mater..

[28] Kuwabara K., Horii F. (1999). Solid-state 13C NMR analyses of the orthorhombic-to-hexagonal phase transition for constrained ultradrawn polyethylene fibers. Macromolecules.

[29] Kitamura R., Horii F., Murayama K. (1986). Erratum: Phase structure of lamellar crystalline polyethylene by solid-state high-resolution 13C NMR: Detection of the crystalline-amorphous interphase (Macromolecules (1986) 19, 3, (636)). Macromolecules.

[30] Gulmine J.V., Janissek P.R., Heise H.M., Akcelrud L. (2002). Polyethylene characterization by FTIR. Polym. Test..

[31] Kaneko M., Kumagai S., Nakamura T., Sato H. (2004). Study of sulfonation mechanism of low-density polyethylene films with fuming sulfuric acid. J. Appl. Polym. Sci..

[32] Grassi A., Longo P., Guerra G. (1989). Solid-state high-resolution 13C NMR spectra of syndiotactic polystyrene. Makromol. Chem., Rapid Commun..

[33] Joseph R., Ford W.T., Zhang S., Tsyurupa M.P., Pastukhov A.V., Davankov V.A. (1996). Solid-state 13C-NMR analysis of hypercrosslinked polystyrene. J. Polym. Sci. A. Polym. Chem..

[34] Coughlin J.E., Reisch A., Markarian M.Z., Schlenoff J.B. (2013). Sulfonation of polystyrene: Toward the “ideal” polyelectrolyte. J. Polym. Sci. Part A Polym. Chem..

[35] Jalal N.M., Jabur A.R., Hamza M.S., Allami S. (2020). Sulfonated electrospun polystyrene as cation exchange membranes for fuel cells. Energy Rep..

[36] Tabekh H., Al Kurdi M.H., Ajji Z. (2015). Sulphonation of expanded polystyrene waste with commercial sulphuric acid for potential use in removal of heavy metals from contaminated waters. Polimeri.

[37] Noritake A., Hori M., Shigematsu M., Tanahashi M. (2008). Recycling of polyethylene terephthalate using high-pressure steam treatment. Polym. J..

[38] Al-Sabagh A.M., Yehia F.Z., Eshaq G., Rabie A.M., ElMetwally A.E. (2016). Greener routes for recycling of polyethylene terephthalate. Egypt. J. Pet..

[39] Yang W., Liu R., Li C., Song Y., Hu C. (2021). Hydrolysis of waste polyethylene terephthalate catalyzed by easily recyclable terephthalic acid. Waste Manag..

[40] Chinchillas-Chinchillas M.J., Orozco-Carmona V.M., Alvarado-Beltrán C.G., Almaral-Sánchez J.L., Sepulveda-Guzman S., Jasso-Ramos L.E., Castro-Beltrán A. (2019). Synthesis of recycled poly(ethylene terephthalate)/polyacrylonitrile/styrene composite nanofibers by electrospinning and their mechanical properties evaluation. J. Polym. Environ..

[41] Valh J.V., Vončina B., Lobnik A., Zemljič L.F., Škodič L., Vajnhandl S. (2020). Conversion of polyethylene terephthalate to high-quality terephthalic acid by hydrothermal hydrolysis: The study of process parameters. Text. Res. J..

[42] Bunn A., Cudby M.E.A., Harris R.K., Packer K.J., Say B.J. (1982). High resolution 13C n.m.r. spectra of solid isotactic polypropylene. Polymer (Guildf).

[43] Morent R., De Geyter N., Leys C., Gengembre L., Payen E. (2008). Comparison between XPS- and FTIR-analysis of plasma-treated polypropylene film surfaces. Surf. Interface Anal..

[44] Tada H., Ito S. (1997). Conformational change restricted selectivity in the surface sulfonation of polypropylene with sulfuric acid. Langmuir.

