# Supplementary material for: Effect of Chemical Agents on the Morphology and Chemical Structures of Microplastics
Source: Polymers (Basel). 2022 Oct 15;14(20):4353. doi: 10.3390/polym14204353 (PMC9609594; doi:10.3390/polym14204353)
Supplement: Supplementary file 1 [file polymers-14-04353-s001.zip › polymers-1955071-supplementary.pdf]

Supplementary data

Effect of Chemical Agents on the Morphology and Chemical Structures of Microplastics

Hak Bong Lee[a], Kyong Sub Lee[a], Seok Jun Kim[b], Byung Il Choi[b], Byung Rye Go[c], Chan Joo Rhu[d], and Tae Hee Han[a,e*]

[a]Department of Organic and Nano Engineering, Hanyang University, Seoul 04763, Republic of Korea

[b]Materials & Components Research Institute, Korea Testing & Research Institute, Gyeonggi-do 13810, Republic of Korea

[c]Products Conformity Center, Korea Conformity Laboratories, Seoul 06711, Republic of Korea

[d]Consumer Product Center, Korea Conformity Laboratories, Seoul 06711, Republic of Korea

[e]The Research Institute of Industrial Science, Hanyang University, Seoul 04763, Republic of Korea

*Corresponding Authors

Prof. Tae Hee Han (T. H. Han)

E-mail: than@hanyang.ac.kr

Keywords: microplastic, chemical resistance, chemical digestion, degradation

Keywords: microplastic, strong acid, chemical resistance, polymer, chemical digestion

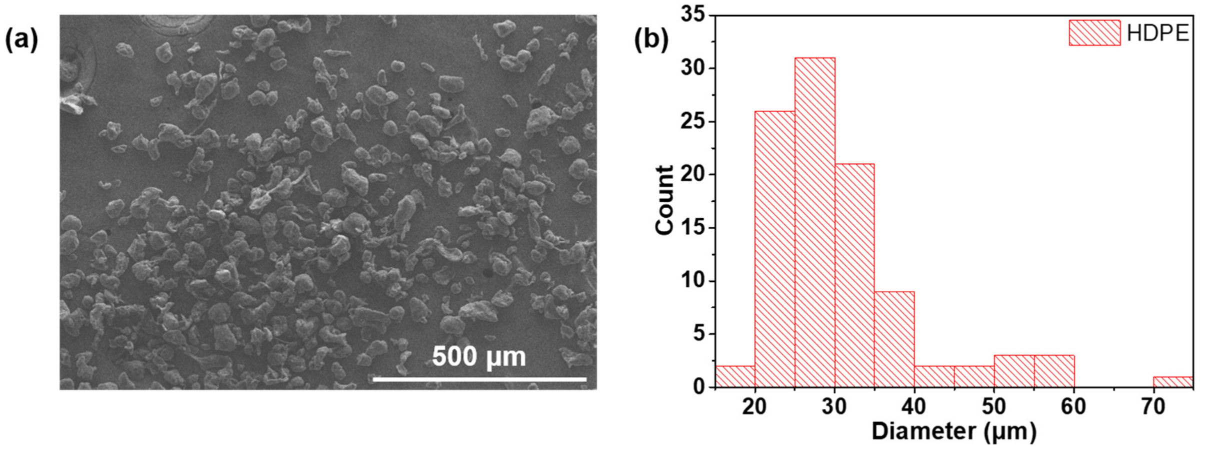

Figure S1. (a) SEM image of the HDPE MP model samples and (b) size-distribution graph.

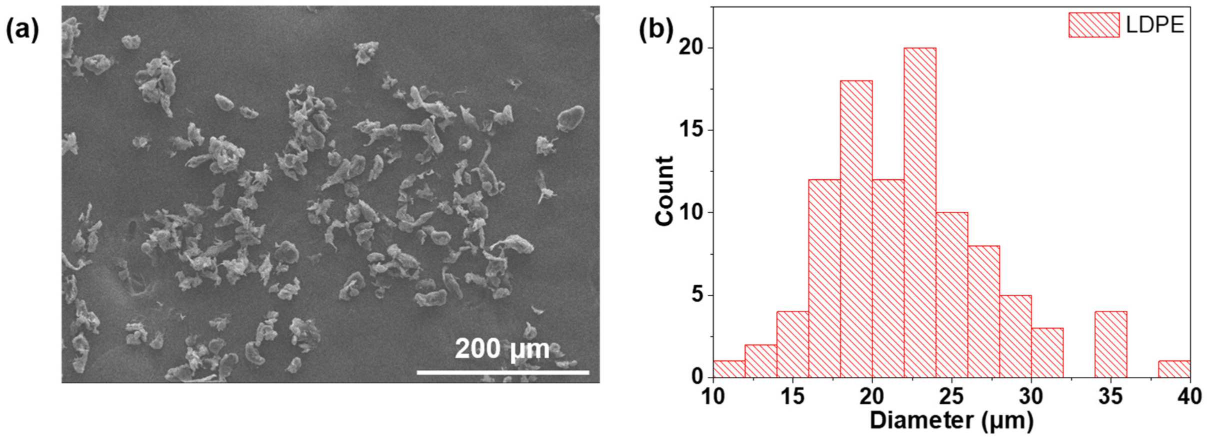

Figure S2. (a) SEM image of the LDPE MP model samples and (b) size-distribution graph.

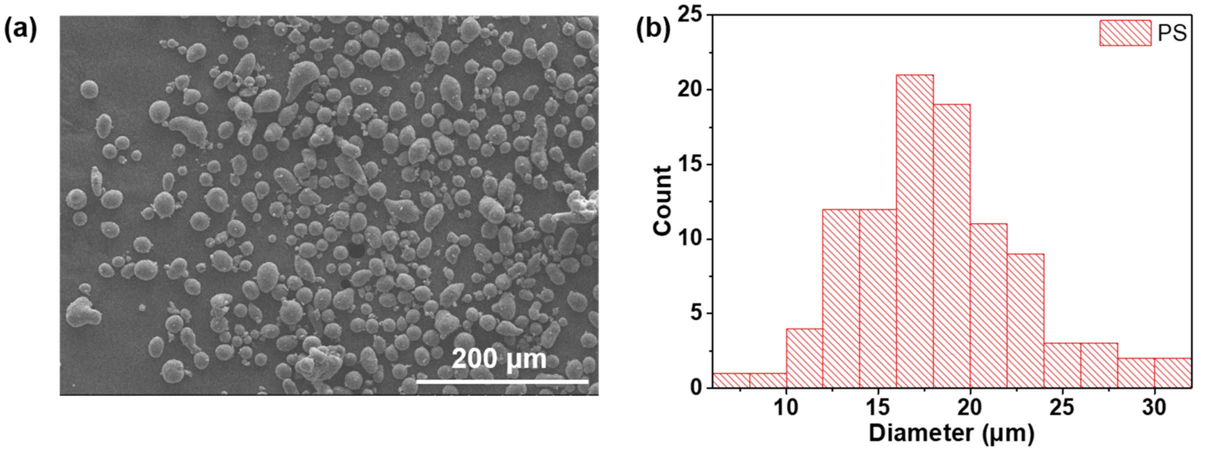

Figure S3. (a) SEM image of the PS MP model samples and (b) size-distribution graph.

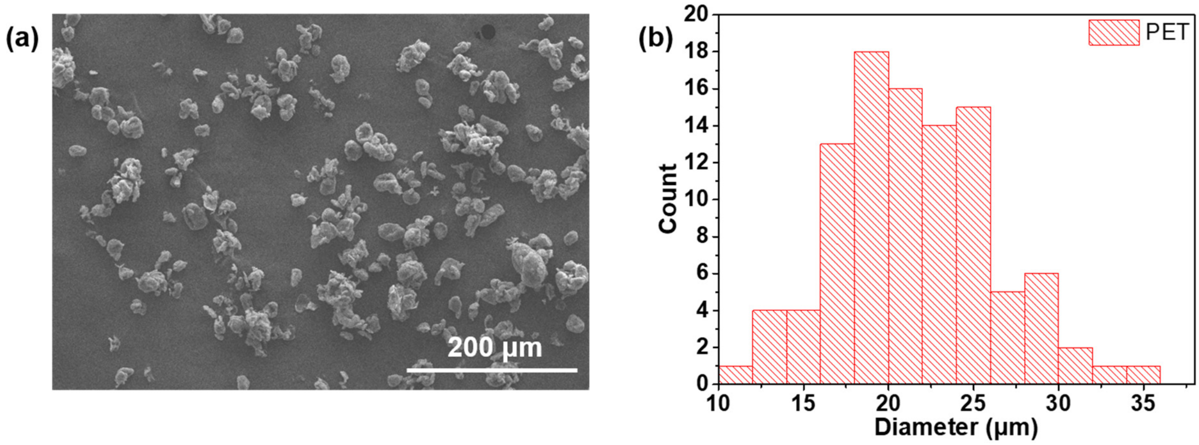

Figure S4. (a) SEM image of the PET MP model samples and (b) size-distribution graph.

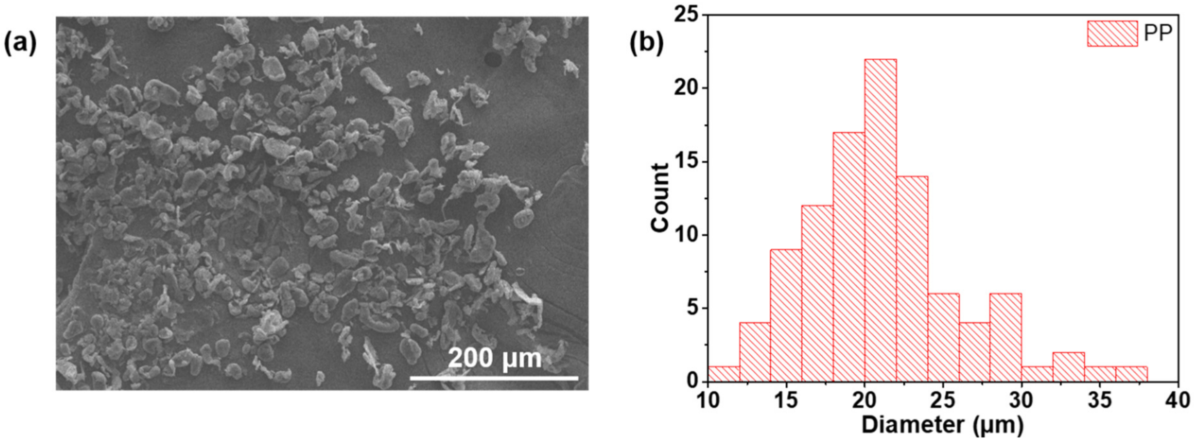

Figure S5. (a) SEM image of the PP MP model samples and (b) size-distribution graph.

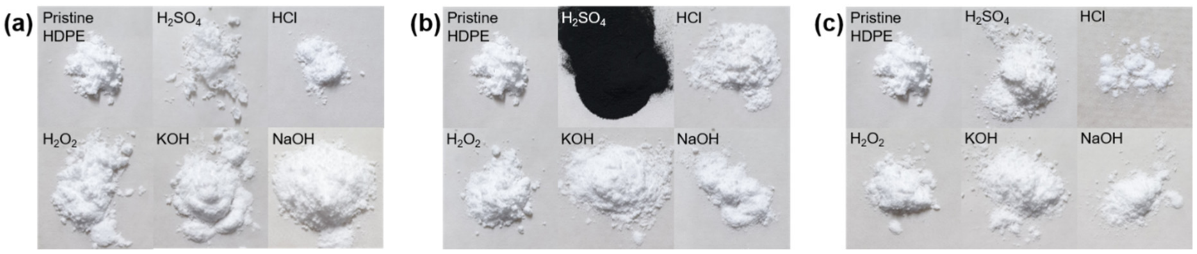

Figure S6. Photographs of the HDPE samples in the pristine state, following chemical stability tests against strong acids and alkalis (H_2SO_4, HCl, H_2O_2, KOH, and NaOH) used in the chemical digestion procedure at (a) 25 °C for 1 d, (b) 70 °C for 1 d, and (c) 25 °C for 7 d.

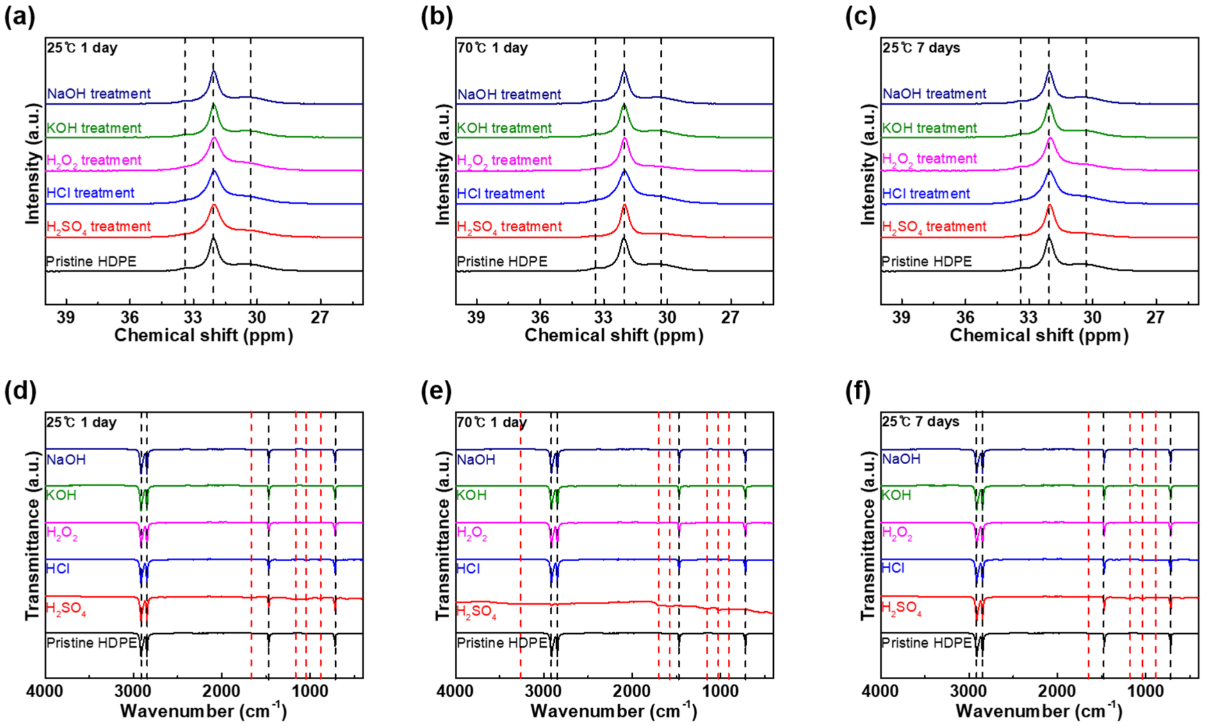

Figure S7. ^{13}C NMR spectra of pristine HDPE and the HDPE samples after reaction with H_2SO_4, HCl, H_2O_2, KOH, and NaOH at (a) 25 °C for 1 d, (b) 70 °C for 1 d, and (c) 25 °C for 7 d. FT-IR spectra of pristine HDPE and the HDPE samples after reaction with H_2SO_4, HCl, H_2O_2, KOH, and NaOH at (d) 25 °C for 1 d, (e) 70 °C for 1 d, and (f) 25 °C for 7 d.

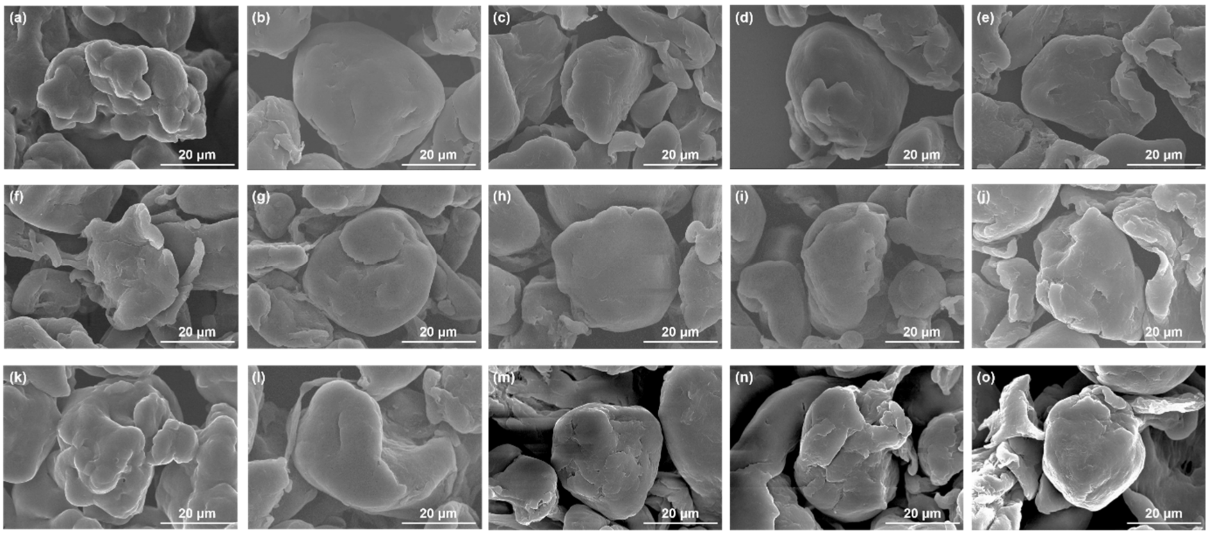

Figure S8. SEM images of the HDPE samples after reaction with (a) H_2SO_4, (b) HCl, (c) H_2O_2,

(d) KOH, and (e) NaOH at 25 °C for 1 d. SEM images of the HDPE samples after reaction with (f) H_2SO_4, (g) HCl, (h) H_2O_2, (i) KOH, and (j) NaOH at 70 °C for 1 d. SEM images of the HDPE samples after reaction with (k) H_2SO_4, (l) HCl, (m) H_2O_2, (n) KOH, and (o) NaOH at 25 °C for 7 d.

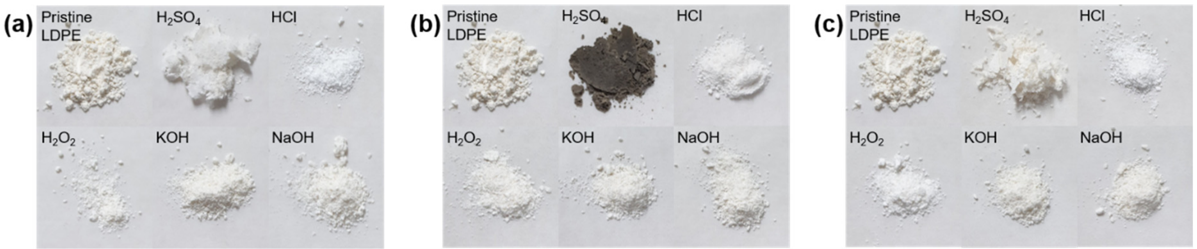

Figure S9. Photograph of the LDPE samples in the pristine state and after undergoing chemical stability tests against strong acids and alkalis at (a) 25 °C for 1 d, (b) 70 °C for 1 d, and (c) 25 °C for 7 d.

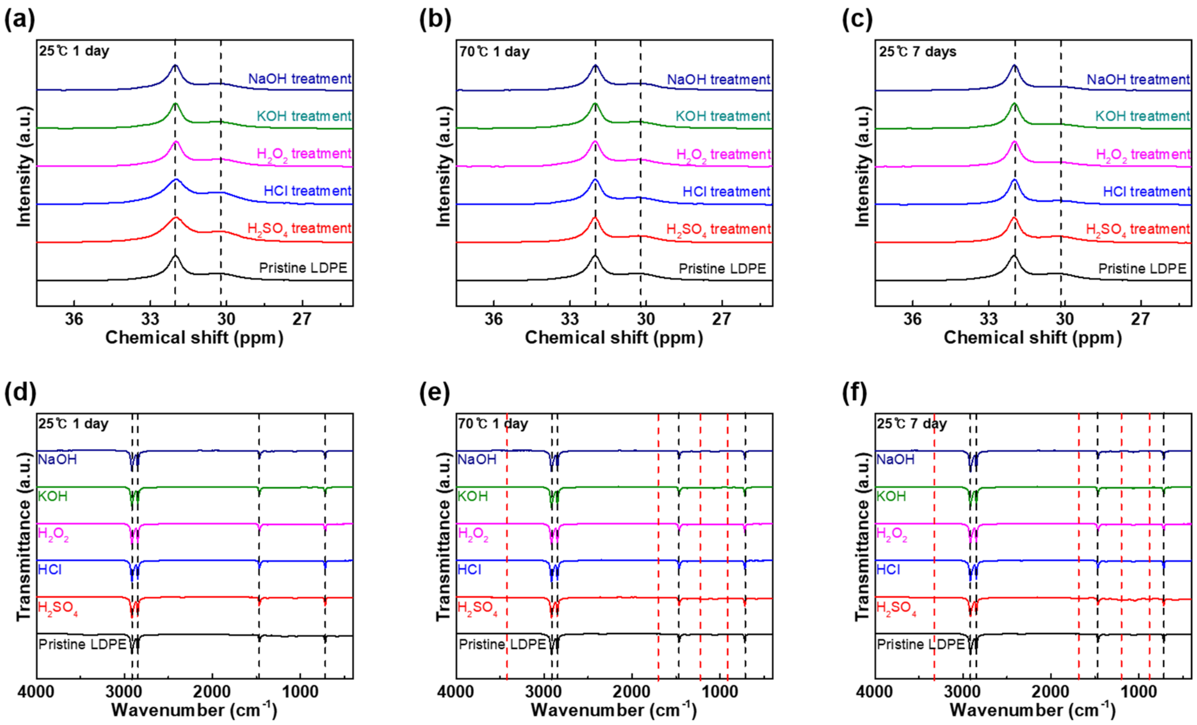

Figure S10. ^{13}C NMR spectra of pristine LDPE and the LDPE samples after reaction with H_2SO_4, HCl, H_2O_2, KOH, and NaOH at (a) 25 °C for 1 d, (b) 70 °C for 1 d, and (c) 25 °C for 7 d. FT-IR spectra of pristine LDPE and the LDPE samples after reaction with H_2SO_4, HCl,

H_2O_2, KOH, and NaOH at (d) 25 °C for 1 d, (e) 70 °C for 1 d, and (f) 25 °C for 7 d.

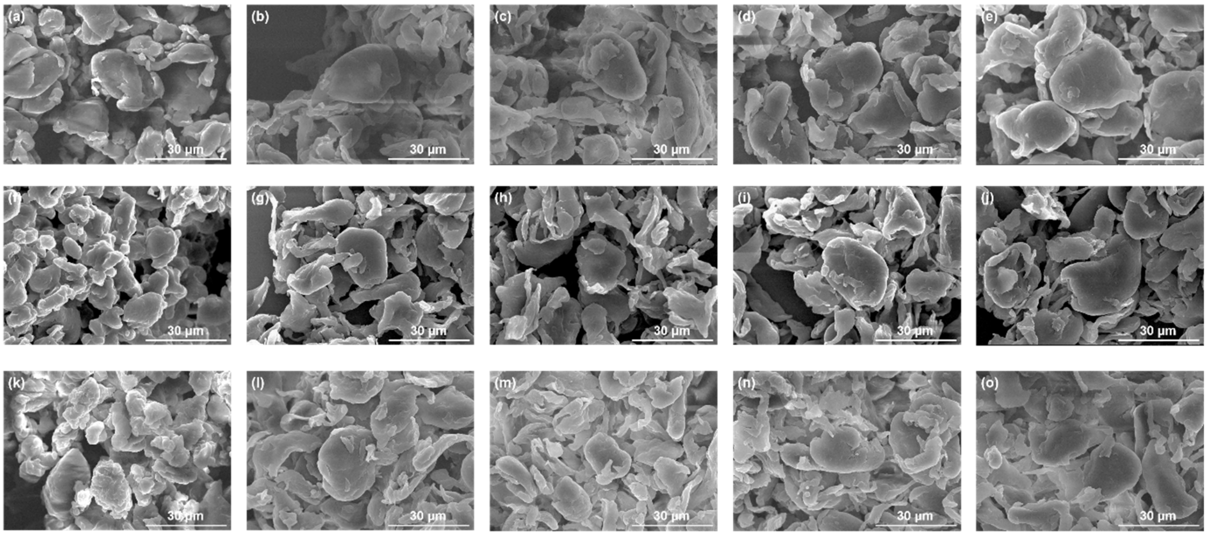

Figure S11. SEM images of the LDPE samples after reaction with (a) H_2SO_4, (b) HCl, (c) H_2O_2, (d) KOH, and (e) NaOH at 25 °C for 1 d. SEM images of the LDPE samples after reaction with (f) H_2SO_4, (g) HCl, (h) H_2O_2, (i) KOH, and (j) NaOH at 70 °C for 1 d. SEM images of LDPE samples after reaction with (k) H_2SO_4, (l) HCl, (m) H_2O_2, (n) KOH, and (o) NaOH at 25 °C for 7 d.

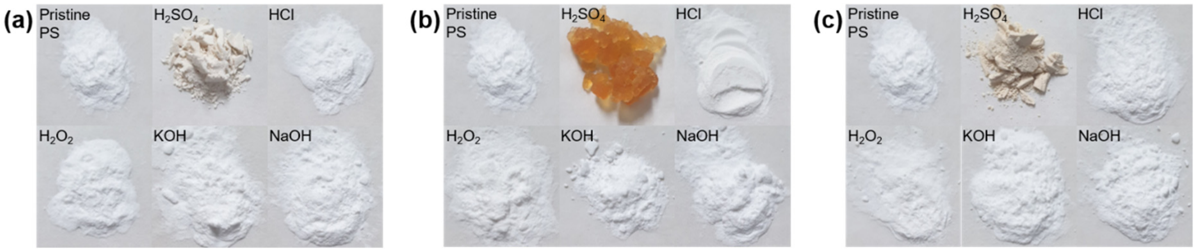

Figure S12. Photograph of the PS samples in the pristine state and after chemical stability tests against strong acids and alkalis at (a) 25 °C for 1 d, (b) 70 °C for 1 d, and (c) 25 °C for 7 d.

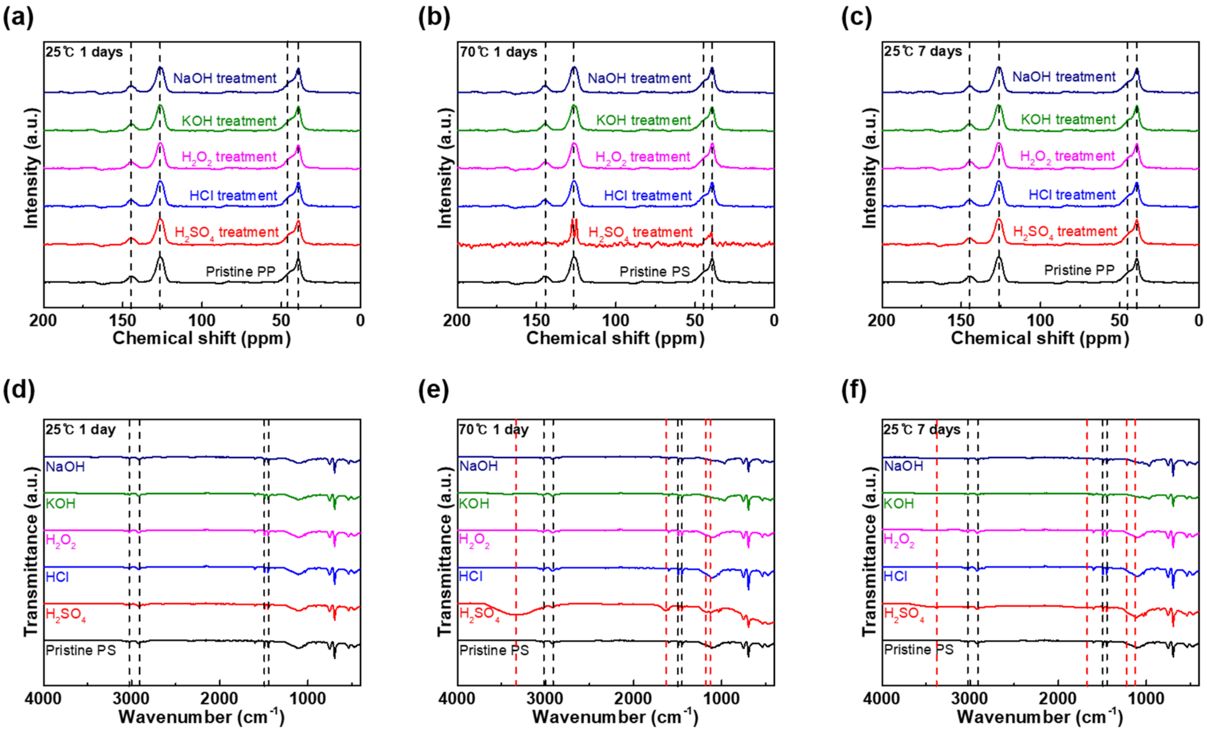

Figure S13. ^{13}C NMR spectra of pristine PS and the PS samples after reaction with H_2SO_4, HCl, H_2O_2, KOH, and NaOH at (a) 25 °C for 1 d, (b) 70 °C for 1 d, and (c) 25 °C for 7 d. FT-IR spectra of pristine PS and the PS samples after reaction with H_2SO_4, HCl, H_2O_2, KOH, and NaOH at (d) 25 °C for 1 d, (e) 70 °C for 1 d, and (f) 25 °C for 7 d.

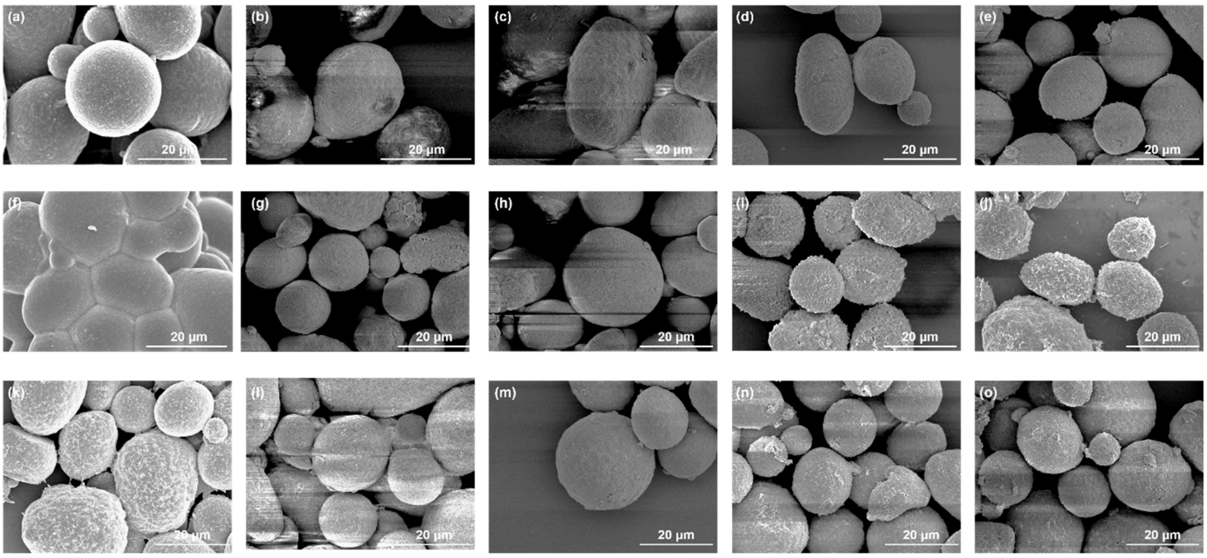

Figure S14. SEM images of the PS samples after reaction with (a) H_2SO_4, (b) HCl, (c) H_2O_2, (d) KOH, and (e) NaOH at 25 °C for 1 d. SEM images of the PS samples after reaction with (f)

H_2SO_4, (g) HCl, (h) H_2O_2, (i) KOH, and (j) NaOH at 70 °C for 1 d. SEM images of the PS samples after reaction with (k) H_2SO_4, (l) HCl, (m) H_2O_2, (n) KOH, and (o) NaOH at 25 °C for 7 d.

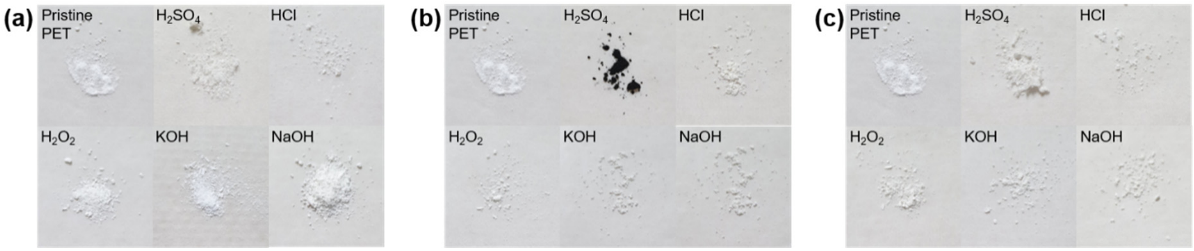

Figure S15. Photograph of the PET samples in the pristine state and after chemical stability tests against strong acids and alkalis at (a) 25 °C for 1 d, (b) 70 °C for 1 d, and (c) 25 °C for 7 d.

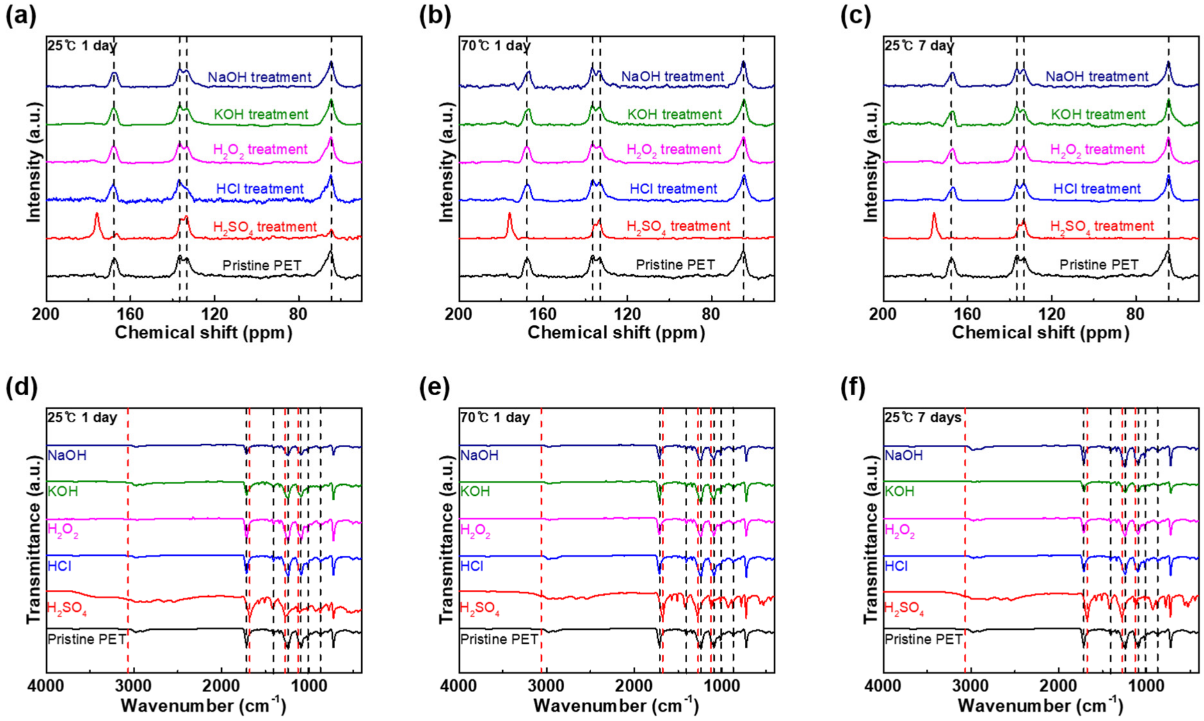

Figure S16. ^{13}C NMR spectra of pristine PET and the PET samples after reaction with H_2SO_4, HCl, H_2O_2, KOH, and NaOH at (a) 25 °C for 1 d, (b) 70 °C for 1 d, and (c) 25 °C for 7 d. FT-IR spectra of pristine PET and the PET samples after reaction with H_2SO_4, HCl, H_2O_2, KOH, and NaOH at (d) 25 °C for 1 d, (e) 70 °C for 1 d, and (f) 25 °C for 7 d.

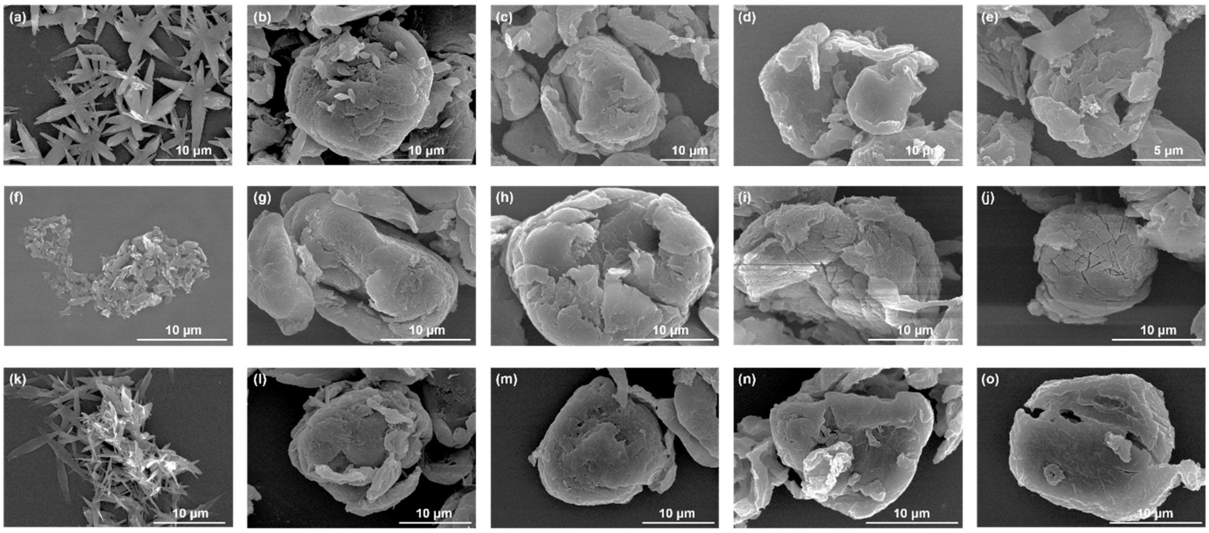

Figure S17. SEM images of the PET samples after reaction with (a) H_2SO_4, (b) HCl, (c) H_2O_2, (d) KOH, and (e) NaOH at 25 °C for 1 d. SEM images of the PET samples after reaction with (f) H_2SO_4, (g) HCl, (h) H_2O_2, (i) KOH, and (j) NaOH at 70 °C for 1 d. SEM images of the PET samples after reaction with (k) H_2SO_4, (l) HCl, (m) H_2O_2, (n) KOH, and (o) NaOH at 25 °C for 7 d.

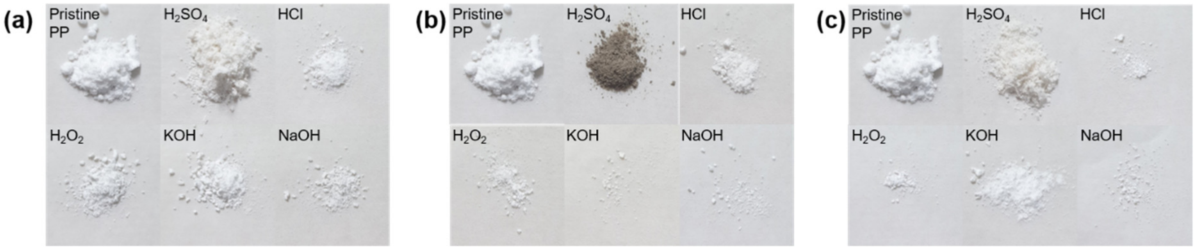

Figure S18. Photograph of the PP samples in the pristine state and after chemical stability tests against strong acids and alkalis at (a) 25 °C for 1 d, (b) 70 °C for 1 d, and (c) 25 °C for 7 d.

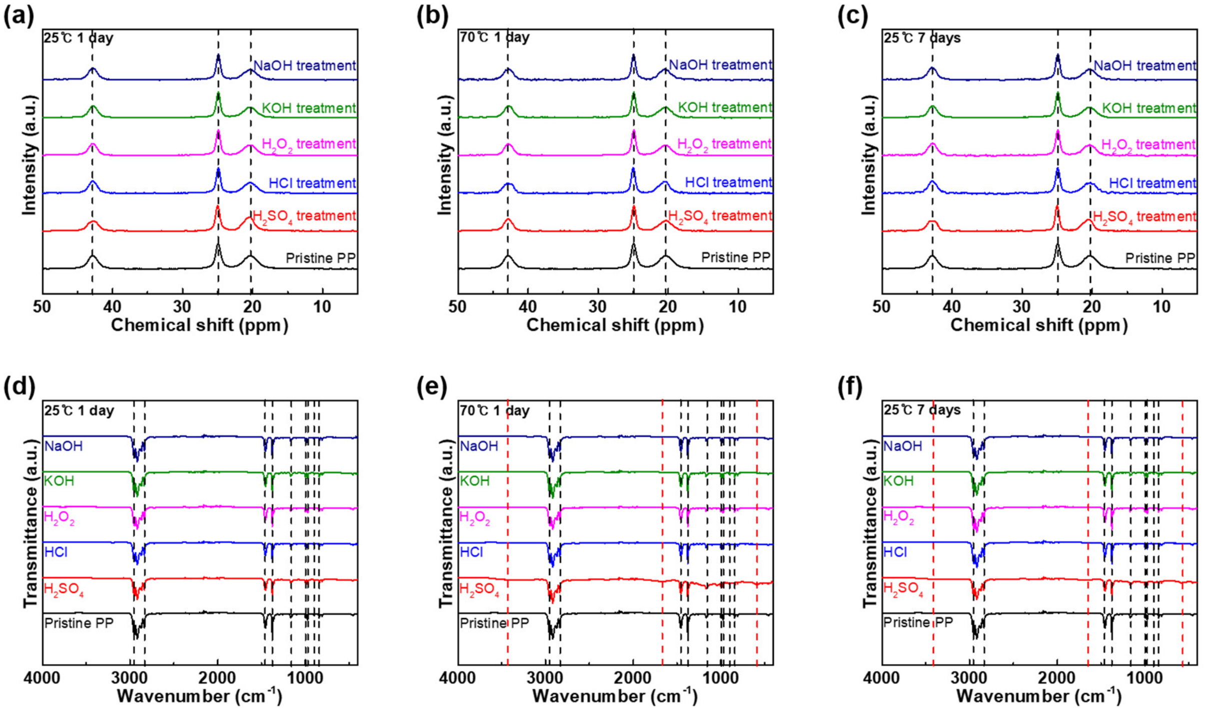

Figure S19. ^{13}C NMR spectra of pristine PP and the PP samples after reaction with H_2SO_4, HCl, H_2O_2, KOH, and NaOH at (a) 25 °C for 1 d, (b) 70 °C for 1 d, and (c) 25 °C for 7 d. FT-IR spectra of pristine PP and the PP samples after reaction with H_2SO_4, HCl, H_2O_2, KOH, and NaOH at (d) 25 °C for 1 d, (e) 70 °C for 1 d, and (f) 25 °C for 7 d.

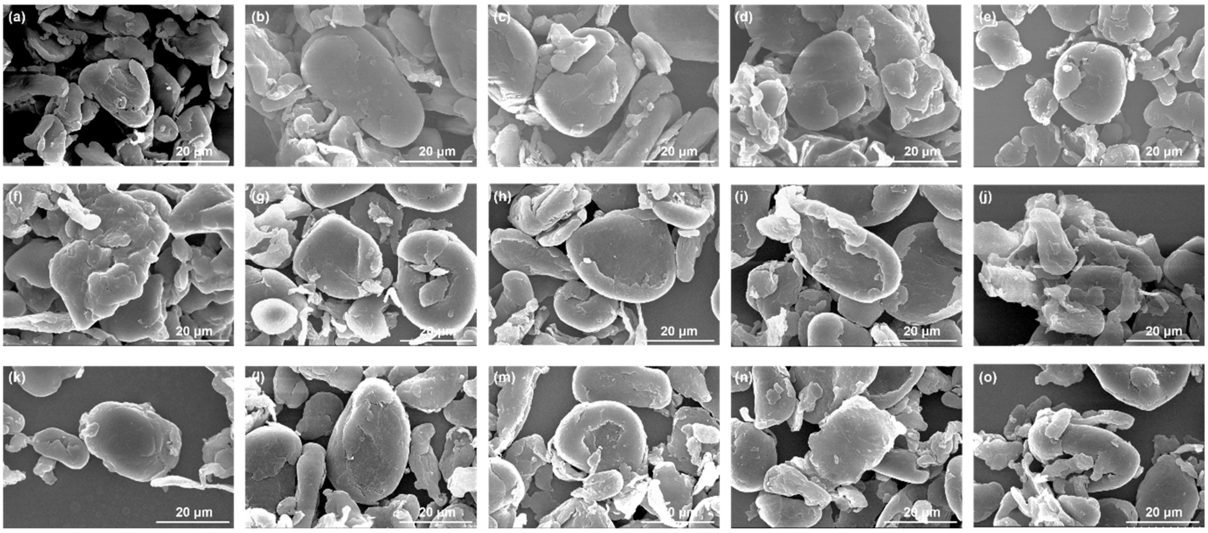

Figure S20. SEM images of the PP samples after reaction with (a) H_2SO_4, (b) HCl, (c) H_2O_2, (d) KOH, and (e) NaOH at 25 °C for 1 d. SEM images of the PP samples after reaction with (f) H_2SO_4, (g) HCl, (h) H_2O_2, (i) KOH, and (j) NaOH at 70 °C for 1 d. SEM images of the PP

samples after reaction with (k) H_2SO_4, (l) HCl, (m) H_2O_2, (n) KOH, and (o) NaOH at 25 °C for 7 d.
